# Clinical Spectrum of Dopa-Responsive Dystonia and Related Disorders

**DOI:** 10.1007/s11910-014-0461-9

**Published:** 2014-05-22

**Authors:** Woong-Woo Lee, Beom Seok Jeon

**Affiliations:** 1 Movement Disorder Center, CRI, Seoul National University Hospital, Seoul, Korea; 2 Department of Neurology, College of Medicine, Seoul National University, Seoul, Korea; 3 Department of Neurology, Seoul National University Hospital, Seoul, Korea

**Keywords:** Dopa-responsive dystonia, DRD-plus, GTP cyclohydrolase I, Tyrosine hydrolase, Dopamine transporter, Vesicular monoamine transporter

## Abstract

Dopa-responsive dystonia (DRD) has a classic presentation of childhood or adolescent-onset dystonia, mild parkinsonism, marked diurnal fluctuations, improvement with sleep or rest, and a dramatic and sustained response to low doses of L-dopa without motor fluctuations or dyskinesias. However, there have been many papers on patients with a wide range of features, which report them as DRD mainly because they had dystonic syndromes with L-dopa responsiveness. Many mutations in the dopaminergic system have been found as molecular genetic defects. Therefore, the clinical and genetic spectra of DRD are unclear, which lead to difficulties in diagnostic work-ups and planning treatments. We propose the concept of DRD and DRD-plus to clarify the confusion in this area and to help understand the pathophysiology and clinical features, which will help in guiding diagnostic investigations and planning treatments. We critically reviewed the literature on atypical cases and discussed the limitations of the gene study.

**Electronic supplementary material:**

The online version of this article (doi:10.1007/s11910-014-0461-9) contains supplementary material, which is available to authorized users.

## Introduction

Dopa-responsive dystonia (DRD) was first recognized by Segawa et al. in 1972 [1]. They described 2 female cousins who showed gait disturbance with dystonia at ages 4 and 6.5. The symptoms were relatively mild in the morning and became severe in the late afternoon or evening. The 2 patients improved markedly by L-dopa treatment.

In fact, Segawa’s report was not the first description for DRD. In 1947, Beck reported an 8.5-year old girl who had showed “kicking up her left heel when walking.” She had generalized dystonia, tremor, masked face, and difficulty in gait. Her paternal uncle had similar problems with walking at age 8. She was reported as “a typical case of dystonia musculorum deformans” [2]. Several years later, the same patient was re-assessed by Corner [3]. He noted that her symptoms waxed and waned, and trihexyphenidyl improved her performance remarkably. These features are in good agreement with what is now known as DRD.

There have been many reports with similar clinical features under different diagnostic terms [2, 4–13]. The term “DRD” was suggested by Nygaard et al. [12, 14], and has generally been accepted. However, there have been many reports under the name DRD with atypical or “incompatible” features as will be discussed below, leading to confusion as to what DRD exactly is. Therefore, in 1998 we proposed a definition for DRD and DRD-plus as a clinical syndrome based on all the information at that time [15, 16]. We believe that this concept clarifies much of the confusion as to the clinical features and helps with the diagnostic work-up and treatment planning. Because the misuse of the term DRD continues, we critically reviewed the literature and recapitulated the concept.

## Clinical Presentation of Classical DRD

Typical clinical features of DRD are childhood or adolescent-onset dystonia sometimes associated with mild parkinsonism, marked diurnal fluctuations, and improvement with sleep or rest, and a dramatic and sustained response to low doses of L-dopa without motor fluctuations or dyskinesias as the hallmark of the disease [17]. Diurnal fluctuations are not specific to DRD, because they could arise in other neurologic disorders [18•, 19, 20]. A marked and sustained response to L-dopa without motor fluctuations or dyskinesias is the most important feature that enables clinicians to distinguish DRD from other dystonias and parkinsonism.

There are many reports of atypical presentations and variable onset age from early infancy to late adulthood. Early infantile presentations mimic cerebral palsy [21]. Adult onset presentations include focal dystonia and parkinsonism [22]. Psychomotor retardation, convulsion, systemic symptoms, and cerebellar dysfunction have also been reported [23, 24]. Even though an excellent response to L-dopa is the hallmark of DRD and delayed treatment for decades has resulted in complete resolution of symptoms [25], an incomplete response for action dystonia has been reported [26]. An autosomal dominant heterozygous mutation in the GTP cyclohydrolase I (GCH-1) gene resulting in biopterin deficiency, which is the cofactor for tyrosine hydroxylase (TH) and dopamine deficiency is the most classic molecular pathophysiology [27].

## Definition of DRD and DRD-Plus

Because of clinical and genetic heterogeneity, there may be confusion as to what DRD is. To prove this point, there have been reports under the name of DRD, which do not fit into the known pathophysiology of DRD, adding confusion in this area. Therefore, we previously proposed that DRD be defined as a syndrome of selective nigrostriatal dopamine deficiency caused by genetic defects in the dopamine synthetic pathway without nigral cell loss [16]. It covers all the typical and atypical presentations of DRD with proven mutations, and allows for a diagnosis of DRD without genetic confirmation.

We further proposed the term "DRD-plus", defined as inherited disorders, in dopamine metabolism, which have features of DRD and additional features that are not seen in DRD [16]. We suggested that this concept is useful in allowing for a diagnosis of DRD without requiring proof of gene mutations, which is not practical and may not be possible in some cases, and in the systematic planning of the clinical and laboratory evaluation of patients who have some features of DRD but who also have features that have not been reported or are unexpected in DRD. The phenotypes of DRD-plus are as follows: (1) earlier onset than DRD such as neonatal onset, (2) more severe motor phenotypes such as poor sucking, swallowing difficulties, severe hypotonia, and (3) nonmotor features (extra-nigrostriatal dopaminergic dysfunctions) such as convulsions (grand mal or myoclonic attacks), psychomotor retardation, mental retardation, drowsiness, irritability, recurrent hyperthermia without infections, and ptosis.

We then predicted that the dichotomy might not always be rigid concerning the causative mutation because the same gene mutation may present as DRD or DRD-plus depending on the degree of the enzymatic deficiency. In the following review, we will emphasize that these heterogeneous groups can be divided into DRD in the strict sense and DRD-plus. This division will help to understand the pathophysiology, clinical features, diagnostic investigation and treatment planning.

## Pathogenesis

**Fig. 1:**
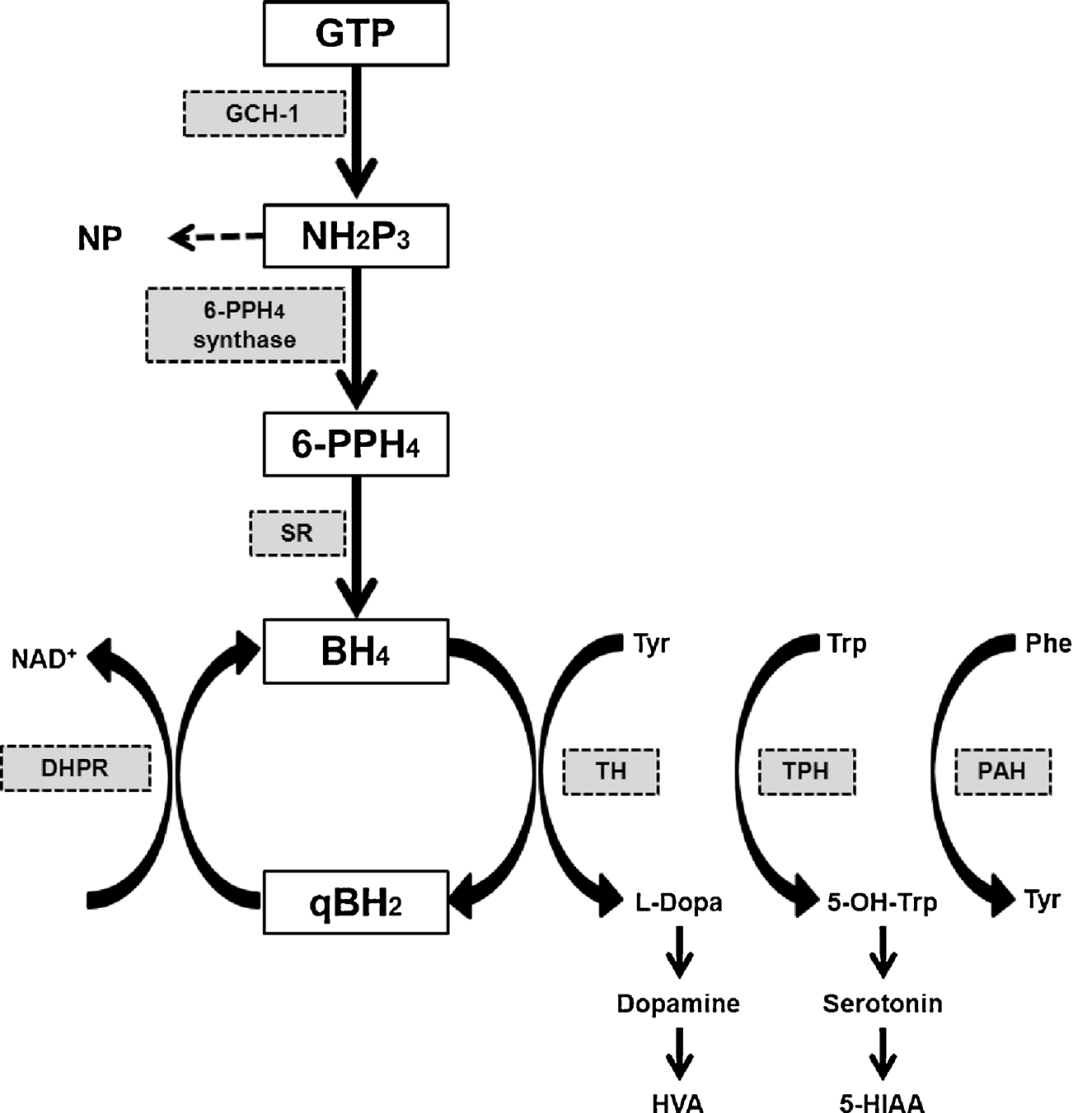
Dopamine biosynthetic pathway

Tetrahydrobiopterin (BH4) is a cofactor of TH. TH makes dopamine from tyrosine. GCH-1 takes the initial step in the synthesis of BH4. The other steps in BH4 metabolism are 6-Pyruvoyltetrahydropterin synthase (6-PPH4 synthase), sepiapterin reductase (SR), and dihydropteridine reductase (DHPR) (Fig. 1). The defect in GCH-1 activity causes decreased dopamine synthesis, and a low neopterin level [28, 29]. On the other hand, the deficiency of 6-PPH4 synthase, SR, or DHPR does not seem to affect the neopterin level.

DRD patients do not show hyperphenylalaninemia (HPA), although BH4 is also a cofactor for phenylalanine hydroxylase [30]. Nigrostriatal dopaminergic neurons appear to be more vulnerable to the BH4-deficient state than that of liver cells. It may be because BH4 stimulates TH gene expression and, thereby, plays a role in the control of the steady-state levels of the protein for which it acts as a cofactor [31]. Another possibility is that GCH-1 in the brain may be differentially regulated from that in the liver. GCH-1 activity in various tissues might depend on different degrees of expression of the normal and mutant mRNAs [29]. It is of interest that brain biopterin loss (−82 %) in asymptomatic carriers was similar with that reported in DRD patients (−84 %), but TH protein and dopamine levels (−52 % and −44 %, respectively) were less severe (DRD patients, −97 % and −88 %, respectively). The results mean that TH protein loss may affect critically the clinical symptoms of DRD [32].

In stark contrast to Parkinson’s disease (PD), pathologic examination shows no degenerative nigral cell loss even though there is a decrease of melanin containing neurons in the pars compacta of the substantia nigra (SN) [33]. The pathologic findings of DRD are supported by fluorodopa positron emission tomography (PET) and dopamine transporter imaging. It presents normal uptake patterns in DRD [15, 34–36].

Our definition of DRD as a selective nigrostriatal dopamine deficiency caused by genetic defects in the dopamine synthetic pathway without nigral cell loss means that DRD is not a neurodegenerative disorder, but a biochemical disorder which should be completely reversed by replacement of the depleted neurochemicals.

## Work-up

### Biochemistry

#### Phenylalanine Loading Test

BH4 is also the cofactor for phenylalanine hydroxylase. Therefore, a defect in BH4 can result in HPA as in cases with autosomal-recessive homozygous or compound heterozygous mutations [24, 37]. Autosomal-dominant GCH-1 deficiency has a selective GCH-1 defect in the brain and not in the liver, thus, does not present with HPA. However, hepatic phenylalanine hydroxylase with partial BH4 deficiency is not able to convert phenylalanine to tyrosine at a normal rate under loading conditions. Profiles of plasma phenylalanine and tyrosine and phenylalanine/tyrosine ratios are abnormal after oral phenylalanine loading [38]. Although this test can differentiate between asymptomatic and symptomatic gene carriers, false-positive, and false-negative results are possible. In fact, heterozygote carriers for phenylketonuria show the same abnormal phenylalanine and tyrosine profiles [39], whereas a small number of genetically confirmed DRD subjects showed no abnormalities with this test [40]. The additional measurement of plasma total biopterin improves the sensitivity [38]. The phenylalanine loading test could help with a differential diagnosis between GCH-1 deficiency and TH deficiency. Phenylalanine blood levels remain increased in GCH-1 deficiency, and are normal in TH deficiency.

#### Neopterin and Biopterin

The phenylalanine loading test is not helpful in distinguishing GCH-1 deficiency from SR deficiency, whereas neopterin and biopterin measurements are useful in discriminating between GCH-1 deficiency and SR deficiency. Neopterin is a catabolic product, which is formed in the first stage of the BH4 synthetic pathway by the GCH-1 enzyme (Fig. 1). Biopterin is a degradation product from BH4 or dihydrobiopterin (BH2), and is found in the last part of the BH4 biosynthesis pathway. Therefore, GCH-1 deficiency shows decreased neopterin and biopterin levels in CSF. In contrast, the CSF neopterin level in SR deficiency is normal while the CSF biopterin level is high [30].

Neopterin in CSF is decreased to less than 20 % of normal values in DRD, which is lower than the values for PD and other dopa-nonresponsive basal ganglia diseases [41]. CSF neopterin in asymptomatic carriers is 30 %–40 % of normal values [42].

#### Enzyme Assay (Measurement of GCH-1)

Enzymatic diagnosis of DRD is limited by the fact that the enzyme GCH-1 is not expressed in blood cells and fibroblasts. An assay in phytohemagglutinin-stimulated mononuclear blood cells shows reduced activity in heterozygous and homozygous GCH-1 patients, and in DRD patients [43, 44]. The activity of GCH-1 in mononuclear blood cells is decreased to less than 20 % of normal levels in patients and to 30 %–40 % in asymptomatic carriers [44]. Cytokine-stimulated fibroblasts are useful for measuring the neopterin and biopterin production patterns and GCH-1 activity. After stimulation with interferon-γ and tumor necrosis factor-α, the concentrations of neopterin and biopterin were extremely low compared with those of normal fibroblasts [45]. There were reports that the abnormal cDNA of abnormal GCH-1 gene produced an enzyme protein without activity [44], and that the proportion of mutant mRNA compared with normal mRNA for GCH-1 was 28 % in the affected patients, and 8 % in asymptomatic carrier [46].

### Imaging Study

DAT is contained in the dopaminergic nerve terminals. DAT imaging reflects the integrity of nigrostriatal dopaminergic nerve terminals. DAT binding in the striatum is markedly decreased in patients with PD and juvenile PD, whereas it is normal in DRD [15, 33, 47–49]. Fluorodopa PET shows similar findings in that fluorodopa uptake is markedly decreased in PD and JPD, whereas it is normal in DRD [34–36]. These results show that there is nigral cell loss and accompanying loss of dopaminergic nerve terminals in PD and JPD, whereas there is no nigral cell loss and intact dopaminergic nerve terminals in DRD. Thus, DAT imaging and fluorodopa PET are extremely useful in differentiating DRD from JPD.

### Gene Study

Because not all GCH-1 deficient DRD patients have GCH-1 mutations in the gene’s coding region or in the splice sites, which are detectable by current genomic DNA sequencing of the GCH-1 gene [50], and because of the high occurrence of sporadic mutations in this gene [51], DNA testing for the autosomal dominant DRD is not suitable for routine clinical practice and must be complemented by enzymatic tests. Suzuki et al. reported that in 40 % of DRD patients no mutation was found in the GCH-1 gene [52]. The inability to detect GCH-1 mutations in apparently typical phenotypes was also reported in the papers of Hagenah et al. and Zirn et al. where extensive screening for GCH-1 was done [53, 54].

## Mutation of the GCH-1 Gene

GCH-1 is a good example of how the same gene can cause different phenotypes such as DRD and DRD-plus depending on the severity of the mutation, which was predicted in the above section. Hahn et al. classified GCH-1 gene mutations into the 3 different phenotypes depending on the gene defects: (1) autosomal dominant hereditary progressive and/or levodopa-responsive dystonia (AD GCH-1 deficiency - the prototype of DRD, characterized by childhood-onset dystonia with sustained clinical responsiveness to low doses of L-dopa); (2) autosomal recessive GCH-1 deficient HPA (AR GCH-1 deficient HPA, presenting in early life with a severe neurologic disorder such as psychomotor retardation, convulsions, etc.); and (3) compound heterozygote mutations (intermediate in severity between the above disorders) [55].

Patients with the AD GCH-1 deficiency have a dystonic movement disorder without mental retardation or convulsions. Biochemically, they have a milder defect in biogenic amine and BH_4_ metabolism (based on the results of cerebrospinal fluid (CSF) studies) than patients with the recessive form of the disease. With L-dopa administration, the motor dysfunctions in these patients dramatically improve. Biochemical studies of patients with recessive mutations of the GCH-1 gene have shown severe defects in BH_4_ metabolism that correlate with the severity of the neurologic symptoms, low biogenic amine metabolite levels in the CSF, and only partial responsiveness to neurotransmitter precursors (L-dopa and 5-hydroxytryptophan) and cofactor administration [24]. Marked depletion of BH4 in brainstem serotonin neurons, which have important roles in postural augmentation and locomotion induces hypotonia and difficulty in locomotive movements or crawling in infancy [56]. Furthermore, the serotonin neurons have important roles in the functional development of the cortex by modulating synaptogenesis [57]. The changes in the non-nigrostriatal dopaminergic system and structural changes exceed the definition of DRD, thus, making these cases DRD-plus, and indicate that symptoms may not be completely reversed by neurochemical replacement therapy.

Furukawa reported intermediate cases between DRD and AR GCH-1 deficient HPA [51]. Patient 1 showed a rather severe motor phenotype of developmental delay and truncal hypotonia at age 6 months, and lethargy at age 5 years. L-dopa did not completely reverse her motor disability. Oral BH4 in Patient 1 provided significant additional benefits, but did not result in complete resolution of her motor problems. Patient 2 had a more benign developmental motor delay of sitting alone at 8 months, crawling at 12 months, and walking independently at 18 months. Language development was normal even though he had mild dysarthria. He was able to walk independently with braces with 150 mg of L-dopa daily. Both cases had compound heterozygous mutations in GCH-1, and had substantially lower BH4 and neopterin levels in the CSF than that of DRD. Therefore, the severity of the BH4 biosynthetic defect in the 2 compound heterozygotes described here appears to be intermediate between that seen in DRD and AR GCH-1 deficient HPA. The authors state that they are intermediate clinically and biochemically between DRD and AR GCH-1 deficient HPA disorders. In our scheme, Patient 1 is regarded as DRD-plus, and Patient 2 may as well be classified as DRD. It is interesting to note that even though both cases had compound heterozygous mutations, Patient 1 had frame shift and missense mutations, whereas patient 2 had 2 missense mutations. We suspect that the mutation in Patient 1 was more severe than that in Patient 2, causing a more severe biochemical deficiency and clinical phenotype. However, the CSF BH4 levels in these cases were similar. Nevertheless, they cannot be directly compared because of the different age at testing and the age-dependent changes in the normal range [58].

Because the residual enzyme activity will determine the phenotype, we suspect that the mild form of homozygous or compound heterozygous mutation in GCH-1 will result in DRD but not DRD-plus as in Patient 2 from Furukawa et al. study [24]. A case by Hwu et al. illustrates this point [59]. The case was a 12-year-old girl with dystonia, diurnal fluctuation, and a sustained good response to L-dopa. She had a homozygous Arg249Ser mutation, and a normal amount of GCH-1 mRNA but low GCH-1 activity. In transfected eukaryotic cells, the Arg249Ser mutant protein expression level was lower than that of the wild-type protein. Therefore, Arg249Ser was suspected to be a destabilizing mutation. A patient with typical DRD was reported as a “compound heterozygote with the AR trait”. The authors reported 2 mutant alleles Lys224Stop and Pro23Leu in the GCH-1. However, 1 mutation Pro23Leu is believed to be functionally insignificant. This case is better regarded as DRD with an AD GCH-1 mutation in 1 allele (Lys224Stop) [60]. These cases emphasize that the severity of mutation and protein dysfunction determines the clinical phenotype rather than the number of mutations and the relevance of the uncovered mutation should be interpreted with care in terms of the clinical presentation.

## Mutations of TH

TH enzymatic activity is mainly expressed in the brain and adrenal medulla. Therefore, measurement of TH enzyme activity in blood cells or cultured fibroblasts is not a diagnostic option. Decreased CSF levels of homovanillic acid (HVA) and 3-methoxy-4-hydroxyphenylglycol (MHPG), together with normal pterin and CSF tyrosine and 5-Hydroxyindoacetic acid (5-HIAA) concentrations are the diagnostic hallmarks of isolated TH deficiency. Clinical severity correlates with the biochemical phenotype, probably depending on the nature of the causative mutations as discussed in GCH-1 [61].

Three phenotypes have been reported: (1) progressive infantile encephalopathy [62, 63], (2) L-Dopa-responsive infantile parkinsonism with a rather good response to L-dopa therapy, which was limited by the occurrence of dyskinesia [64], and (3) typical DRD [65–67].

Progressive infantile encephalopathy is dominated by motor retardation, fluctuating extrapyramidal, and ocular and vegetative symptoms. Treatment with L-dopa ameliorates but usually does not normalize symptoms [63]. L-dopa-responsive infantile parkinsonism is characterized by early infantile onset severe motor disturbances including parkinsonism, myoclonic jerks, and ptosis due to sympathetic denervation. Parkinsonism is responsive to L-dopa and ptosis to an ocular instillation of 2.5 % (w/v) phenylephrine [64]. The above cases belong to DRD-plus in our scheme.

Typical DRD was also described in reports as mentioned below [65–67]. Two siblings manifested with lower-limb onset generalized dystonia. Patient 1 showed walking difficulties with increased muscle tone at age 3, and was in a wheelchair-bound state within 10 years. Patient 2, the younger brother, also had walking problems due to dystonia, which made him wheelchair-bound within 7 years. He revealed developmental delay and panic disorder, probably because of rhesus incompatibility and from the drug treatment, respectively. They had a compound heterozygous mutation (Asp498Gly, Ala376Val). Both had a sustained response to low-dose L-dopa for over 35 years [65]. In another report, 3 patients from 2 families had onset at age 2. At age 5, they were wheelchair bound. L-dopa normalized motor performance, and the benefit continued without fluctuation in efficiency after 30 years [67]. Ludecke et al. reported 2 siblings with moderate extrapyramidal symptoms with clinical onset in the first decade and a dramatic positive therapeutic response to low-dose L-DOPA therapy. Diurnal fluctuations were present. The genetic defect in exon 11 was Gln381Lys. No biochemical assay was done [66].

## Mutations of SR

SR converts 6-PPH4 to BH4. A defect in SR causes dopamine deficiency, serotonin deficiency, and neurotoxicity [30]. Patients with SR deficiency show diurnally fluctuations and dopa-responsive movement disorders like in typical DRD cases. However, most patients have additional neurologic problems, such as developmental delay, hypotonia, oculogyric crises, and cognitive impairment due to additional serotonergic defects [68–74]. CSF analysis revealed low 5-HIAA and HVA and high total biopterin, BH2, and sepiapterin. Neopterin is not significantly different from the reference range. Decreased SR activity in fibroblasts and genetic study are helpful in diagnosis [75].

Patients with SR deficiency show clinical heterogeneity depending on the severity of the genetic and enzymatic defect. A patient with a homozygous mutation presented with mental and growth retardation, microcephaly, and spasticity, as well as dystonia [68]. His SR activity in fibroblasts was under 10 mU/mg (controls, 99 ~ 185 mU/mg). On the other hand, the DRD phenotype was reported in a heterozygous mutation of SR [69]. The patient was a 26-year-old woman who walked on tiptoes since childhood. Motor disturbance was the only manifestation, which showed pronounced fluctuations. L-dopa was effective but with headaches and nausea. The SR activity in her fibroblasts was 62 mU/mg, which is higher than that of the previous homozygous case. Haplo-insufficiency of the sepiapterin reductase gene (*SPR*) was suggested as the molecular pathologic mechanism resulting in DRD in the patient. There is another report showing gene-enzyme-phenotype correlations, in which Arrabal et al. presented 4 cases of SR deficiency and showed a good correlation between the severity of the mutations and the severity of the phenotypes [74]. SR deficiency showed good L-dopa responsiveness in general, and received additional benefits from 5-hydroxytryptophan in cognitive deficit, as well as motor and sleep symptoms [75].

## Mutations of the Dopamine Transporter and Vesicular Monoamine Transporter 2: Transportopathies

There are 2 important reports showing deficits in the neurotransmitter transporting system, not in the biosynthesis of dopamine [76••, 77••]. The mutations of *SLC6A3,* a gene located on 5p15.3, results in DAT deficiency [76••]. Because the function of DAT is mainly reuptake of dopamine into presynaptic dopaminergic neurons, DAT deficiency results in abundant dopamine in the synaptic cleft and overactivation of D2 autoreceptors leading to decreased dopamine production. The clinical feature of DAT deficiency is very similar to DRD-plus as we proposed. The age of onset is younger than that of DRD. It shows nonmotor and systemic manifestations, as well as parkinsonism and dystonia. There are no diurnal fluctuations in DAT deficiency. L-dopa was only partially effective. For the diagnosis, DAT imaging is very helpful, revealing complete loss of DAT binding in the basal ganglia. Vesicular monoamine transporter 2 (VAMT2) deficiency affects more of the neurotransmitter system, including dopamine, serotonin, epinephrine, and norepinephrine [77••]. Jennifer et al. showed that a mutation in *SLC18A2*, a gene encoding VMAT2, causes the defects in monoamine transport. This disorder also shared many clinical characteristics with DRD-plus, although L-dopa was completely ineffective toward parkinsonism and dystonia. The characteristics of DAT deficiency and VAMT deficiency are presented in Table 1.

**Table 1:** Differential diagnosis of JPD, DRD, DRD-plus, and transportopathy

	JPD	DRD	DRD-plus	Transportopathy	
				DAT deficiency	VMAT2 deficiency
Age of onset	Childhood~Adolescence	Childhood~Adolescence	Infancy	Infancy	Infancy
Symptoms and signs					
Motor symptoms					
Dystonia	+/−	+	+/−	+	+
Parkinsonism	+	+/−	+/−	+	+
Nonmotor symptoms	-	-	+	+	+
Systemic symptoms	-	-	+	+	+
Diurnal fluctuation	+/−	+	+/−	-	-
Laboratory tests					
DAT imaging	Abnormal	Normal	N/A^a^	Markedly abnormal	N/A^a^
CSF NTs	Neopterin:	A/T subtype^b^	A/T subtype^b^	HVA/5-HIAA:	Normal
Urine NTs	N/A^a^	A/T subtype^b^	A/T subtype^b^	HVA:	5-HIAA, HVA:
					NE, Dopamine:
Phenylalanine loading test	N/A^a^	A/T subtype^b^	A/T subtype^b^	N/A^a^	N/A^a^
L-dopa response^c^					
Dose	Small^d^	Small	Large	Large	No response
Response degree	Good	Marked	Partial	Partial	No response^e^
Motor complications^f^	Frequent^g^	Absent	Present^h^	Present^h^	Present^h^

^a^It is predicted to be normal.

^b^According to subtype. Please refer to Supplement Table 1 for details.

^c^Dopa agonist is more effective than L-dopa in AADC deficiency, DAT deficiency and VMAT2 deficiency.

^d^Dose increases with time.

^e^Responds to dopamine agonists.

^f^Motor fluctuation and dyskinesias.

^g^Motor complications occur as a late-complication.

^h^Dyskinesia may appear early by the administration of L-dopa in a dose-dependent manner.

*5-HIAA* hydroxyindoleacetic acid, *AADC* Aromatic L-amino acid decarboxylase, *A/T* According to, *DAT* dopamine transporter, *DRD* dopa-responsive dystonia, *HVA* homovanillic acid, *JPD* juvenile Parkinson’s disease, *N/A* not available, *NE* norepinephrine, *NTs* neurotransmitters, *VMAT2* vesicular monoamine transporter 2

## Atypical Cases

Our definition of DRD is selective nigrostriatal dopaminergic deficiency. There could be a long discussion as to what neurologic features can be presented by selective nigrostriatal dopaminergic deficiency; however, here we mean only motor features. Thus, DRD by our definition allows for parkinsonism and dystonia only. However, there have been reports of atypical extranigral phenotypes in “DRD”. We argue that these observations in DRD are just coincidental and not causal.

Depression was not a frequent observation in DRD. Segawa even argued that depression could be coincidental because he only reported 1 depressive person among 28 gene proven patients from 15 families [26]. However, 2 articles emphasized a high prevalence of psychiatric manifestations. Hahn et al. described 11 mutation cases from among more than 70 members in the pedigree with a high prevalence of depression (4 of 11) and anxiety (6 of 11) [55]. However, the authors did not examine mutation noncarriers in the family to compare with mutation carriers raising the issue of observation bias and other comorbid disorders in the family. Of interest is that 4 of the 6 cases with anxiety had deafness, again raising the suspicion of comorbid disorders. A study by Hove et al. also highlighted that major depressive disorder and obsessive-compulsive disorder were strikingly more frequent than observed in the general population among mutation carriers in their 3 families with proven GCH-1 deficiency [78]. Patients responded well to serotoninergic medication and to L-dopa substitution. Sleep disorders were present in 55 % of the patients. However, mutation negative members of these families were not examined again raising the issue of observation bias and other comorbid disorders in the family.

Tic syndrome (TS) was seen in 2 siblings with DRD [79]. Both parents also had tics. Because TS did not show diurnal fluctuations and no improvement in the tics was observed, the TS appears to be coincidental.

Even cerebellar dysfunction was described in 2 of 4 genetically confirmed cases [23]. The authors describe horizontal gaze-evoked nystagmus, limb incoordination, and gait ataxia. However, the clinical description of the 4 cases is typical DRD, and not that of ataxic syndrome. This paper also raised the issue of a false-positive test for anti-glutamic acid decarboxylase antibody (patient 3).

## Considerations for Diagnosis

### L-Dopa Responsiveness

Based on the pathophysiology of DRD in our proposed definition, it is expected that low dose L-dopa should completely reverse any symptoms in DRD. However, there are papers on focal dystonia reported as DRD not showing such a remarkable improvement [80, 81]. There are also several cases with proven GCH-1 mutations, showing partial improvement (from no response to 90 % response) [26]. It could be conjectured that a prolonged dopamine deficiency state in the basal ganglia somehow deranged the proper motor circuit resulting in dystonia, which no longer responds to L-dopa. We would like to speculate that these focal dystonias that do not respond completely to L-dopa may not be related to DRD and that the uncovered genetic defect is just coincidental. We would like to note that the penetrance of the GCH-1 mutation is only 30 % supporting that the presence of a mutation does not guarantee clinical symptoms and a diagnosis of DRD [14], and that L-dopa response is so excellent in the most severely affected DRD cases after many years of treatment delay [25] that it is hard to imagine that mild clinical symptoms may be the only and residual symptoms. In our recent report of 19 cases of GCH-1 mutation positive DRD, only 1 case (Case III: 2 in family D) had residual symptoms with facial grimacing and upper limb dystonia [82•]. Of interest is that her brother (case III:1) had isolated torticollis, which did not respond to L-dopa. His gene test revealed no mutation, which raises the possibility of a second familial dystonic disorder affecting Case III:1 and Case III:2. Therefore, it is quite surprising that Tardic et al. [83•] reported high frequencies of residual motor signs in patients receiving therapy in 28 % from a literature review and 39 % from their pilot study group.

There are cases of unusual phenotypes such as torticollis or camptocormia, which are reported as DRD simply because they responded well to L-dopa. A case by Gerpen et al. was a 48-year-old man with progressive camptocormia particularly after exertion [84]. The patient could walk backwards and had sensory tricks. L-dopa (600 mg/day with carbidopa) provided consistent benefits for over 5 years. The unusual points in this patient for DRD are the rather high dose of L-dopa and a rapid recurrence of symptoms after skipping medication. No genetic or biochemical study was done.

There was a very intriguing report about familial torticollis with good dopa-responsiveness [85]. Genetic testing for GCH-1, TH, and SR was negative, and there was no laboratory evidences supporting DRD, such as CSF neurotransmitters and its metabolites, and enzymatic activities. Recently, the authors reported that the 3 affected members had compound heterozygous mutations in *ATM* on chromosome 11 [86•]. Two other patients with the mutation in the family showed typical presentations of ataxia telangiectasia. Sanders-Pullman et al. [87] reported primary-appearing dystonia in Canadian Mennonites who carry mutations in the ATM. L-dopa was not effective in the 3 cases tested. Therefore, the causal relation between the mutations in *ATM* and L-dopa responsiveness (which is the main crux of the term DRD by our definition) appears to be doubtful.

### Mutation-Negative DRD

There were families in which no abnormalities in the gene were detected in the coding region even though there was a linkage to 14q or a decrease of CSF neopterin or GCH 1 activity in peripheral mononuclear blood cells. Recently, in 1 of these families, a defect in the mRNA was observed in 1 allele [88]. DYT14 was initially thought to exclude GCH-1, however, later studies showed a large exon deletion of the GCH-1 gene [89]. In our own paper in 1998, low CSF neopterin suggested a low activity of the GCH-I enzyme in Family C (Patients 6 and 7) and Patient 9 [15]. However, we failed to find mutations in GCH-1 at the time of the report. A recent analysis showed that Family C has exon1 P95R (nt284C > G) and Patient 9 (S4 in Lee et al., 2013) has Gly203Arg [82•]. In addition, a case by Nagata et al. is a perfect example of DRD-plus with a GCH-1 mutation suspected but not found [90]. This 20-year-old woman presented with motor and mental disturbances since ages 2 and 3, respectively. CSF neopterin was extremely low; however, a mutation in GCH-1 was not found. A decrease in serum neopterin, biopterin, and serotonin suggested a severe deficiency in GCH-1. A further search of GCH-1 and its unknown cofactor is warranted based on these laboratory findings. These cases show that genetic study alone has limitations. A biochemical analysis of the CSF would have led the investigation to focus on individual enzymes in BH4 metabolism and TH based on the neopterin level and other chemical levels.

### Coexistence with Degenerative Parkinsonism

There are 4 reported cases of DRD and defective nigrostriatal dopaminergic integrity, which should not be seen in DRD by our definition. The first case is a Danish male presenting with DRD at age 28 but later showed features of young-onset PD (YOPD) at age 35 by manifesting dyskinesia [91]. A mutation in GCH-1 (Pro199Ser) and decreased GCH-1 enzyme activity were found supporting a diagnosis of DRD. However, development of dyskinesia and decreased DAT binding are indicative of YOPD. Therefore, this case should be regarded as a mixture of DRD and YOPD, where YOPD would determine his clinical course. The second case is a 54-year-old man with parkinsonism caused by an Arg184His mutation in GCH-1 [92]. Arg184His was the first mutation causing both the AR and AD phenotypes [92, 93]. Fluctuations and dyskinesia, which are indicative of PD, developed. Fluorodopa PET was abnormal, supporting the diagnosis of PD but not DRD. Therefore, this case should be regarded as a mixture of DRD and PD, where PD would determine his clinical outcome. The third case is on a 76-year-old woman with a 19 year history of parkinsonism [26]. The parkinsonian symptoms had been improved by small doses of L-dopa. However, dyskinesia and dementia had developed subsequently. Her brain image showed an enlarged ventricle and an old stroke lesion. She became wheelchair-bound at age 80 with a fixed flexor posture of the upper limbs and severe dystonic hand deformities. Although her sons showed DRD manifestation with a GCH-1 mutation and she also had a GCH-1 mutation, the diagnosis of DRD is doubtful in terms of the delayed onset, motor complications, dementia, and abnormalities on the brain scan. The last case is a 65-year-old woman with mild parkinsonian features and a complete deletion of the GCH-1 gene on 1 allele [94]. Her [^123^I] FP-CIT single photon emission computed tomography (SPECT) showed bilateral asymmetric putaminal deficit. Although the patient’s symptoms were mild and responded well to low-dose L-dopa, it was slowly progressive. It means that the cause of her problems is a degenerative disease, not an enzymatic defect. Because the prognosis will be determined by degenerative parkinsonism, it will be more reasonable to consider degenerative parkinsonism than DRD in these cases even though mutations in the GCH-1 gene were found in these patients. All these cases emphasize that detailed biochemical analysis, clinical evaluation, DAT imaging and follow-up are needed to be sure that the uncovered genetic defect is the sole cause of their symptoms.

### Differential Diagnosis from JPD

The difficulties of differentiating JPD from DRD are well known, and have been a problem from the outset. Several early clinical descriptions of DRD later turned out to be JPD and vice versa [12, 34, 95–97]. It is quite understandable in that both conditions present as young-onset parkinsonism and dystonia, have a similar remarkable early good therapeutic response to L-dopa and a positive family history. Diurnal fluctuations, even prominent ones, have been a common finding in JPD. Previous reports of DRD, which were later shown to be JPD based on a later appearance of motor fluctuations and dyskinesia, demonstrate this difficulty [12, 95–97]. The brain of 1 patient was even reported twice, with diverse conclusions in 2 separate publications; although the final diagnosis was JPD [98], the patient was initially reported as DRD [34].

Because JPD is neurodegenerative condition whereas DRD is a neurochemical disorder, the long-term clinical course is different in that JPD requires a substantially larger intake of L-dopa and will ultimately develop motor fluctuations and dyskinesias. Because the long-term prognosis is different, an early differential diagnosis is important and possible. The CSF neopterin level and gene studies can be helpful. However, a mild decrease in CSF neopterin is possible even in JPD, and a gene study can be a false negative. DAT imaging or Fluorodopa PET will be the best tests to clearly differentiate the 2 conditions [15, 33–36, 47–49].

## Diagnosis of DRD and DRD-Plus

The concepts of DRD and DRD-plus are much closer to their pathogenesis, and are more practical in diagnosis and treatment. (Table 1 and Supplement Table 1) A good example is a paper by Clot et al. who reported a molecular genetic study on patients under the categories of pure DRD or DRD-plus [99]. Their definitions of pure DRD and DRD-plus were the same as ours even though the authors failed to mention our reports [15, 16]. Among 57 patients with pure DRD, GCH-1, and PARK2 defects were proven in 46 (80.7 %) and 1 (1.8 %) patients, respectively. DAT imaging would have easily selected the patient with PARK2 prior to going through the rigorous genetic screening. The number of patients with DRD-plus syndrome was seven. The results of the genetic screening were as follows: GCH-1 deficiency, 1 case (14.3 %); TH deficiency, 3 cases (42.9 %); SR deficiency, 2 cases (28.6 %). These results show that our definition could reflect genetic causes and is useful in guiding a diagnostic work-up.

**Fig. 2:**
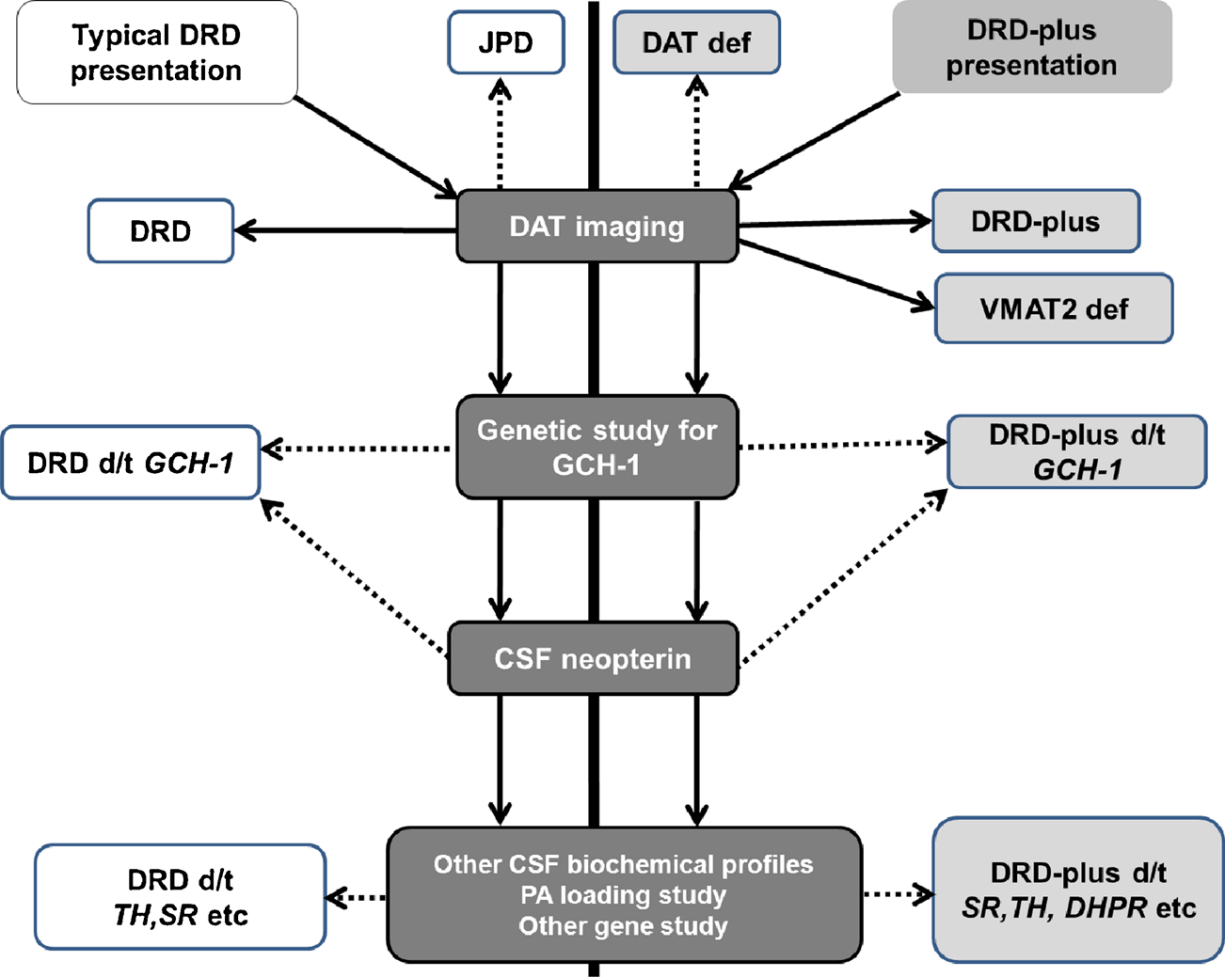
Diagnostic flow for dopa-responsive dystonia and related disorders

If the clinical diagnosis is DRD, then genetic screening of GCH-1 is the most productive (Fig. 2). If GCH-1 screening is negative, the CSF neopterin and biopterin levels will guide the investigation to the individual enzymes in BH4 metabolism and TH. If the CF neopterin is low, an exhaustive study of GCH-1 is needed, whereas a normal CSF neopterin level will necessitate examination of other genes in BH4 metabolism and TH but not in GCH-1. A similar diagnostic algorithm is also logical in DRD-plus. DAT imaging will reliably screen DRD-mimicking JPD. Of note is the recent discovery of DAT and VMAT deficiency syndromes [76••, 77••]. These rare AR genetic disorders have DRD-plus features of hypo- and hyperkinetic movement disorder and ocular motility problems with onset in infancy. The DAT deficiency syndrome had raised ratios of HVA to 5-HIAA in the CSF. DAT imaging showed a complete loss of DAT activity in the basal nuclei. If the patient with the characteristics of DRD-plus has no abnormalities of the neurotransmitters and metabolites in the CSF, the diagnosis of that patient could be VMAT2 deficiency [77••]. VMAT2-deficient patients show abnormal neurotransmitters and metabolites only in the urine.

We maintain that all symptoms of DRD should be completely reversed by small dose of L-dopa. Symptoms of DRD-plus may not be completely reversed because of more extensive non-nigrostriatal, nondopaminergic and structural changes. DRD-plus strongly suggests more severe depletion of BH4 and extranigral nondopaminergic, especially serotonergic involvement. Thus, a BH4 and serotonin trial will be of value.

## Conclusions

Dystonic syndromes with L-dopa responsiveness are very heterogeneous with various clinical, genetic, and biochemical features. Our clinically oriented concept and definition of DRD and DRD-plus will help in understanding the pathophysiology and clinical features, and will help in guiding the diagnostic investigation and treatment planning. A genetic study helps significantly in making the diagnosis. However, it should be noted that the severity of the mutation and protein dysfunction regardless of the causative gene determines the clinical phenotype rather than the presence of mutations, and the same gene mutation may present as DRD or DRD-plus depending on the severity of the enzymatic defect. Some pitfalls in genetic studies such as false-negatives, false-positives and the presence of comorbid conditions will need to be considered in terms of clinical presentation.

## Electronic supplementary material

Below is the link to the electronic supplementary material.

Supplement Table 1 (DOC 42 kb)
