# Supplementary material for: Clinical Spectrum of Dopa-Responsive Dystonia and Related Disorders
Source: Curr Neurol Neurosci Rep. 2014 May 22;14(7):461. doi: 10.1007/s11910-014-0461-9 (PMC4061475; doi:10.1007/s11910-014-0461-9)
Supplement: Supplementary file 1 — (DOC 42 kb) [file 11910_2014_461_MOESM1_ESM.doc]

**Supplement Table1. The profiles of neurotransmitters and its derivatives according to subtypes**

| **Subtype (references)** | **CSF** | **Urine** | **Phenylalanine-loading test** |
| --- | --- | --- | --- |
| **GCH-1 deficiency [1, 2]** | neopterin, biopterin, 5-HIAA, HVA : decrease  BH2, HVA/5-HIAA : normal | HVA, 5-HIAA, VMA, NE, E, dopamine : normal | Positive |
| **TH deficiency [1, 3]** | HVA/5-HIAA : decrease 5-HIAA : normal | HVA, NE : decrease  E/NE : increase | Normal |
| **SR deficiency [2, 4-7]** | HVA, 5-HIAA : decrease  total biopterin, BH2, sepiapterin : increase | neopterins : normal  biopterin : decrease | Positive |
| **DHPR deficiency [8, 9]** | BH4, dopamine, HVA, serotonin, 5-HIAA : decrease  BH2 : increase  neopterin : increase or normal | neopterin, biopterin : increase | Positive |
| **AADC deficiency [10-12]** | HVA, 5-HIAA : decrease  3-OMD : increase | vanillactic acid : increase  VMA, 5-HIAA : decrease | N/A |

GCH-1, GTP cyclohydrolase I; TH, tyrosine hydroxylase; SR, sepiapterin reductase; DHPR, dihydropteridine reductase; AADC, Aromatic L-amino acid decarboxylase; HVA, homovanillic acid; 5-HIAA, hydroxyindoleacetic acid; VMA, vanillylmandelic; NE, norepinephrine; BH2, dihydrobiopterin; BH4, tetrahydrobiopterin; 3-OMD, 3-O-methyldopa

1. Hoffmann GF, Assmann B, Brautigam C, Dionisi-Vici C, Haussler M, de Klerk JB et al. Tyrosine hydroxylase deficiency causes progressive encephalopathy and dopa-nonresponsive dystonia. Annals of neurology. 2003;54 Suppl 6:S56-65. doi:10.1002/ana.10632.

2. Blau N, Bonafe L, Thony B. Tetrahydrobiopterin deficiencies without hyperphenylalaninemia: diagnosis and genetics of dopa-responsive dystonia and sepiapterin reductase deficiency. Molecular genetics and metabolism. 2001;74(1-2):172-85. doi:10.1006/mgme.2001.3213.

3. Brautigam C, Steenbergen-Spanjers GC, Hoffmann GF, Dionisi-Vici C, van den Heuvel LP, Smeitink JA et al. Biochemical and molecular genetic characteristics of the severe form of tyrosine hydroxylase deficiency. Clinical chemistry. 1999;45(12):2073-8.

4. Arrabal L, Teresa L, Sanchez-Alcudia R, Castro M, Medrano C, Gutierrez-Solana L et al. Genotype-phenotype correlations in sepiapterin reductase deficiency. A splicing defect accounts for a new phenotypic variant. Neurogenetics. 2011;12(3):183-91. doi:10.1007/s10048-011-0279-4.

5. Abeling NG, Duran M, Bakker HD, Stroomer L, Thony B, Blau N et al. Sepiapterin reductase deficiency an autosomal recessive DOPA-responsive dystonia. Molecular genetics and metabolism. 2006;89(1-2):116-20. doi:10.1016/j.ymgme.2006.03.010.

6. Echenne B, Roubertie A, Assmann B, Lutz T, Penzien JM, Thony B et al. Sepiapterin reductase deficiency: clinical presentation and evaluation of long-term therapy. Pediatric neurology. 2006;35(5):308-13. doi:10.1016/j.pediatrneurol.2006.05.006.

7. Friedman J, Hyland K, Blau N, MacCollin M. Dopa-responsive hypersomnia and mixed movement disorder due to sepiapterin reductase deficiency. Neurology. 2006;67(11):2032-5. doi:10.1212/01.wnl.0000247274.21261.b4.

8. Sedel F, Ribeiro MJ, Remy P, Blau N, Saudubray JM, Agid Y. Dihydropteridine reductase deficiency: levodopa's long-term effectiveness without dyskinesia. Neurology. 2006;67(12):2243-5. doi:10.1212/01.wnl.0000249335.35585.3e.

9. Blau N, Thony B, Renneberg A, Penzien JM, Hyland K, Hoffmann GF. Variant of dihydropteridine reductase deficiency without hyperphenylalaninaemia: effect of oral phenylalanine loading. Journal of inherited metabolic disease. 1999;22(3):216-20.

10. Hyland K, Surtees RA, Rodeck C, Clayton PT. Aromatic L-amino acid decarboxylase deficiency: clinical features, diagnosis, and treatment of a new inborn error of neurotransmitter amine synthesis. Neurology. 1992;42(10):1980-8.

11. Abdenur JE, Abeling N, Specola N, Jorge L, Schenone AB, van Cruchten AC et al. Aromatic l-aminoacid decarboxylase deficiency: unusual neonatal presentation and additional findings in organic acid analysis. Molecular genetics and metabolism. 2006;87(1):48-53. doi:10.1016/j.ymgme.2005.09.007.

12. Manegold C, Hoffmann GF, Degen I, Ikonomidou H, Knust A, Laass MW et al. Aromatic L-amino acid decarboxylase deficiency: clinical features, drug therapy and follow-up. Journal of inherited metabolic disease. 2009;32(3):371-80. doi:10.1007/s10545-009-1076-1.
